# Hospital readmissions in children with new‐onset infantile epileptic spasms syndrome

**DOI:** 10.1002/epi4.12711

**Published:** 2023-03-30

**Authors:** Chellamani Harini, Christopher J. Yuskaitis, Avantika Singh, Trevor McHugh, Shanshan Liu, Michelle DeLeo, Nishtha Gupta, Candice Marti, Bo Zhang, Mark H. Libenson, Jay G. Berry

**Affiliations:** ^1^ Division of Epilepsy and Clinical Neurophysiology, Department of Neurology Boston Children's Hospital, Harvard Medical School Boston Massachusetts USA; ^2^ Biostatistics and Research Design Center, Institutional Centers for Clinical and Translational Research Boston Children's Hospital, Harvard Medical School Boston Massachusetts USA; ^3^ Department of Neurology Boston Children's Hospital, Harvard Medical School Boston Massachusetts USA; ^4^ Complex Care, Division of General Pediatrics Boston Children's Hospital, Harvard Medical School Boston Massachusetts USA

**Keywords:** hospitalizations, infantile epileptic spasms syndrome, readmissions

## Abstract

**Objective:**

To describe inpatient resource use in the 2 years following infantile epileptic spasms syndrome (IESS) diagnosis, examine the association between clinical/demographic variables and incidence of readmission, and identify risk factors/reasons for frequent readmissions.

**Methods:**

Retrospective cohort analysis of readmissions (scheduled/unscheduled) within the first 2 years following IESS diagnosis, details of readmissions (number/time between rehospitalizations, and length of stay), demographic/clinical variables, and reasons for readmissions were collected. Negative binomial regression analysis evaluated associations between incidence of readmissions (both scheduled/unscheduled and unscheduled alone) and demographic/clinical factors. Logistic regression assessed the risk of having recurrent readmissions (≥5 readmissions).

**Results:**

Among 93 (60% males) new‐onset IESS patients, there were 394 readmissions (56% scheduled and 44% unscheduled) within 2‐years following IESS diagnosis. Mean length of stay was 3.5 days (SD: 5.9). Readmissions occurred in 82 patients (88%) and 37 (40%) experienced ≥5 readmissions. On multivariate regression analysis, readmissions were increased with use of multiple first‐line treatments for IESS (*P* = 0.006), technology assistance (*P* ≤ 0.001), and multispecialty care (*P* = 0.01); seizure freedom (*P* = 0.015) and known etiology (*P* = 0.011) lowered the incidence of readmissions. Examining unscheduled readmissions separately, increased readmissions occurred with public insurance (*P* = 0.013), technology use (*P* ≤ 0.0.001), and multispecialty care (*P* = 0.013); seizure freedom decreased unscheduled readmissions (*P* = 0.006). Technology assistance (G‐tube, NG tube, VP shunt, and tracheostomy use) increased the odds (*P* = 0.007) for recurrent readmissions. Reasons for readmissions included EEG monitoring (protocol driven for verification of IESS remission/characterization of events/EEG surveillance/presurgical monitoring) (51%), acute medical issues (21%), and seizure exacerbation (15%). Protocol‐driven readmissions declined an estimated 52% following protocol modification during the study.

**Significance:**

In the 2 years following IESS diagnosis, there is substantial inpatient resource use with nearly 40% experiencing ≥5 readmissions (mostly epilepsy related). Since readmissions are increased by intrinsic patient characteristics such as medical complexity (technology use and multispecialty care) or epilepsy‐related issues, the preventability of readmissions is uncertain, except for protocol‐driven ones.

**Key Points**

- In the 2 years following IESS diagnosis, there is substantial inpatient resource use, with nearly 40% experiencing ≥5 readmissions.
- Most readmissions are epilepsy related and scheduled readmissions (56%) are more frequent than unscheduled (44%) ones.
- Medical complexity/technology assistance increased frequency of readmissions; public insurance use increased unplanned readmissions only.
- Readmissions included EEG monitoring (planned and unplanned) (51%), acute medical issues (21%), and seizure exacerbation (15%).
- Protocol‐driven inpatient diagnostic admissions decreased by an estimated 52% with protocol modification.

## 1 BACKGROUND

West syndrome, the triad of (a) epileptic spasms, (b) hypsarrhythmia on EEG, and (c) developmental stagnation or regression,^1^ has a high risk for intractable epilepsy, autism, and intellectual impairment.^2^ Infantile epileptic spasms syndrome (IESS) includes infants with epileptic spasms with or without West syndrome.^3^

Following IESS diagnosis, infants are at risk for multiple hospitalizations. During a 2‐year follow‐up of Medicaid beneficiaries with new‐onset IESS, 77% had at least one emergency department (ED) visit or hospitalization.^4^ In another study, IESS patients had a mean of 2.25 hospitalizations in the 1‐year following ACTH initiation with a mean length of stay of 4.2 days per hospitalization.^5^ Following IESS diagnosis, readmissions occur for initiating adrenocorticotrophic hormone (ACTH) therapy,^6^ managing adverse effects of high‐dose hormonal therapy,^7^ protocol‐driven overnight EEG monitoring to assess IESS remission,^8^ or assessing relapses. When first‐line therapies fail to achieve IESS freedom,^9^ alternative therapies are pursued—some of which require hospitalization, including ketogenic diet initiation and epilepsy surgery.^10^, ^11^

Epilepsy patients utilize more health resources than those with other chronic health conditions.^12^, ^13^ Children with uncontrolled epilepsy require more hospitalizations than adults with epilepsy.^14^, ^15^ Compared to other epilepsies, IESS patients have the highest mean annual resource utilization, including days hospitalized.^13^

While some studies have recognized the magnitude of IESS medical burden,^4^, ^5^ the impact of a new diagnosis of IESS on inpatient resource use remains poorly understood. Hospital admissions represented the highest percentage of the total costs in childhood epilepsy, particularly after a new diagnosis.^15^ Understanding risk factors/reasons for readmissions and inpatient resource utilization in IESS is critical to provide parental counseling and insight into potential opportunities to minimize readmissions. Hospitalization data are vital for estimating cost of illness in a target population such as IESS patients. Multiinstitutional databases can provide information regarding hospitalizations: however, these studies have inherent limitations related to lack of granularity in patient‐level data. In contrast, clinical studies tracking individual patients can provide accurate information on reasons/types of readmissions, seizure control, and patient characteristics that could affect hospitalization.

We conducted a retrospective study of hospitalizations in IESS patients with goals to (a) examine inpatient resource use (readmissions and length of stay) in the 2 years following IESS diagnosis, (b) examine factors that lead to increased frequency of readmissions, including identification of risk factors for frequent readmissions (stratified by readmissions frequency), and (c) evaluate reasons for readmissions.

## 2 METHODS

This is an IRB‐approved retrospective cohort analysis of readmissions in the first 2 years following the diagnosis of IESS (index admission).

### 2.1 Study design, setting, and patient population

Children born between January 2014 and June 2019 and had new‐onset IESS, diagnosed between 2 months and 2 years of age with a minimum follow‐up of 2 years, at Boston Children's Hospital (BCH) were included. Patients were excluded if they lacked 2‐year follow‐up, were deceased, or were referred to BCH for a second opinion.

IESS patients were identified from the institutional data warehouse for IESS‐specific diagnosis codes (ICD‐9 codes: 345.60 and 345.61; or ICD‐10 codes: G40.821, G40.822, G40.823, and G40.824). We verified this list against BCH clinical note search utility (“Hound Dog”) searching for “infantile spasms,” “ACTH,” “prednisolone,” and “vigabatrin” through notes within the study's date range (2014‐2019).

### 2.2 Main outcome measures

Hospital readmission (number, timing, and reasons) was the main outcome measure. We included all‐cause readmissions (scheduled/unscheduled) affiliated with any medical or surgical service within the first 2 years following index admission. Data collected included number of readmissions, time between readmissions, and total length of stay.

The primary reason for readmission was determined from chart review (TM). Two reviewers (CH and AS) independently reviewed 50% of charts verifying reasons for readmissions; discrepancies were resolved through discussion. Reasons for readmissions were categorized as scheduled or unscheduled readmissions.

Scheduled readmissions primarily included presurgical evaluations, elective procedures, ketogenic diet initiation, protocol‐driven overnight EEG monitoring for verifying electroclinical remission of IESS, and EEG monitoring for surveillance. Prior to November 2017, our protocol suggested inpatient overnight EEG monitoring to confirm electroclinical remission for patients who were spasm free within 2 weeks following first‐line IESS therapy. This practice is similar to other academic institutions across the United States and in the proposed protocol for treatment of IESS.^7^, ^8^, ^16^ Following November 2017, our protocol shifted to outpatient prolonged EEG around day 14 to confirm electroclinical remission for most spasm‐free patients, with few exceptions (those with history of isolated or subtle spasms).

Reasons for unscheduled readmissions included: acute medical issues, seizure exacerbation, and other unanticipated hospitalizations. Seizure exacerbation needs urgent attention to prevent seizure escalation/status epilepticus. The unpredictable nature/urgency makes this type of readmission an unplanned one.

EEG monitoring for characterization of events (epileptic/nonepileptic) was categorized either as scheduled (admitted via treating neurologist) or unscheduled (admitted via ED). Admission via neurologist or ED depended upon level of clinical concern by the clinician and/or family. When multiple reasons for readmission were noted, the dominant symptom necessitating admission/treatment was chosen as the reason for readmission.

### 2.3 Independent variables

We collected demographic data (age, sex, and insurance type) and IESS‐related information (history of seizures prior to IESS onset, age at onset/diagnosis of IESS, time to initial first‐line treatment, developmental delay at IESS onset based on clinician impression or developmental assessment, IESS etiology (acquired, nonacquired, and unknown), and number of first‐line treatments tried). Further data collection included: use of the ketogenic diet, seizure status at last follow‐up (seizure free for 1 year vs continuing seizures), use of technology assistance (described below), and involvement of multispecialty care (≥3 specialists outside of Pediatrics and Neurology).

First‐line therapies included ACTH, prednisolone, and vigabatrin.^1^, ^17^ If epileptic spasms and/or hypsarrhythmia are not controlled with the initial first‐line medications, then patients may use more than one first‐line medication to treat IESS. Technology assistance was defined as a medical device to maintain a child's health including gastrostomy tube (g‐tube), tracheostomy tube, cerebrospinal fluid ventricular shunt, permanent indwelling catheter,^18^ and nasogastric tube (NG‐tube) when used for nutrition. Insurance types were categorized as: (a) public, (b) private or dual (both private and public), and (c) unknown.

### 2.4 Statistical analysis

Descriptive statistical analysis (count, percentage, mean, standard deviation, median, and interquartile range), univariate, and multivariate generalized linear regression were performed. Negative binomial regression analysis (see Appendix A, Ref. 19) evaluated the association between the number of readmissions in the first 2 years following the diagnosis of IESS and demographic/clinical factors. Incidence rate ratios (IRRs; see Appendix A, Ref. 20) for rehospitalizations in the first 2 years following IESS diagnosis were estimated for variables with 95% confidence intervals (CIs) and corresponding *P*‐values. An IRR of <1 indicated that the incident rate was lower in one group compared to another group, and vice versa. For example, an IRR of 0.88 would indicate a 12% reduction in rate of hospitalization when comparing the group in the numerator to that of the denominator. The number of protocolized readmissions pre‐/postprotocol change was compared using a Chi‐square test.

Following IESS diagnosis, mean emergency room visit/hospitalization rates of 1.6‐3.5 per person per year^4^ and 2.2 in the 1st year of onset have been reported.^5^ Among infants with bronchopulmonary dysplasia and complex‐care program patients, mean rehospitalization rates were 2.2 ± 1.9 in the 1st year of life and 3.1 ± 2.8 over a 2‐year period, respectively.^18^, ^21^ Mean hospitalization readmission rates in our cohort are similar. Therefore, we used our sample mean to define frequent readmissions: patients having ≥5 readmissions (above the mean of our sample) over 2 years following IESS diagnosis. We performed logistic regression to calculate odds ratios (ORs), 95% CIs, with *P*‐values for having ≥5 readmissions in 2 years. Variables were selected using forward selection (entry significance level of 0.2) in multivariate negative binomial and logistic regression analysis without adjusting for multiple comparisons. SAS version 9.4 (SAS Institute, Inc., Cary, NC, USA) was used for analyses.

## 3 RESULTS

**FIGURE 1:**
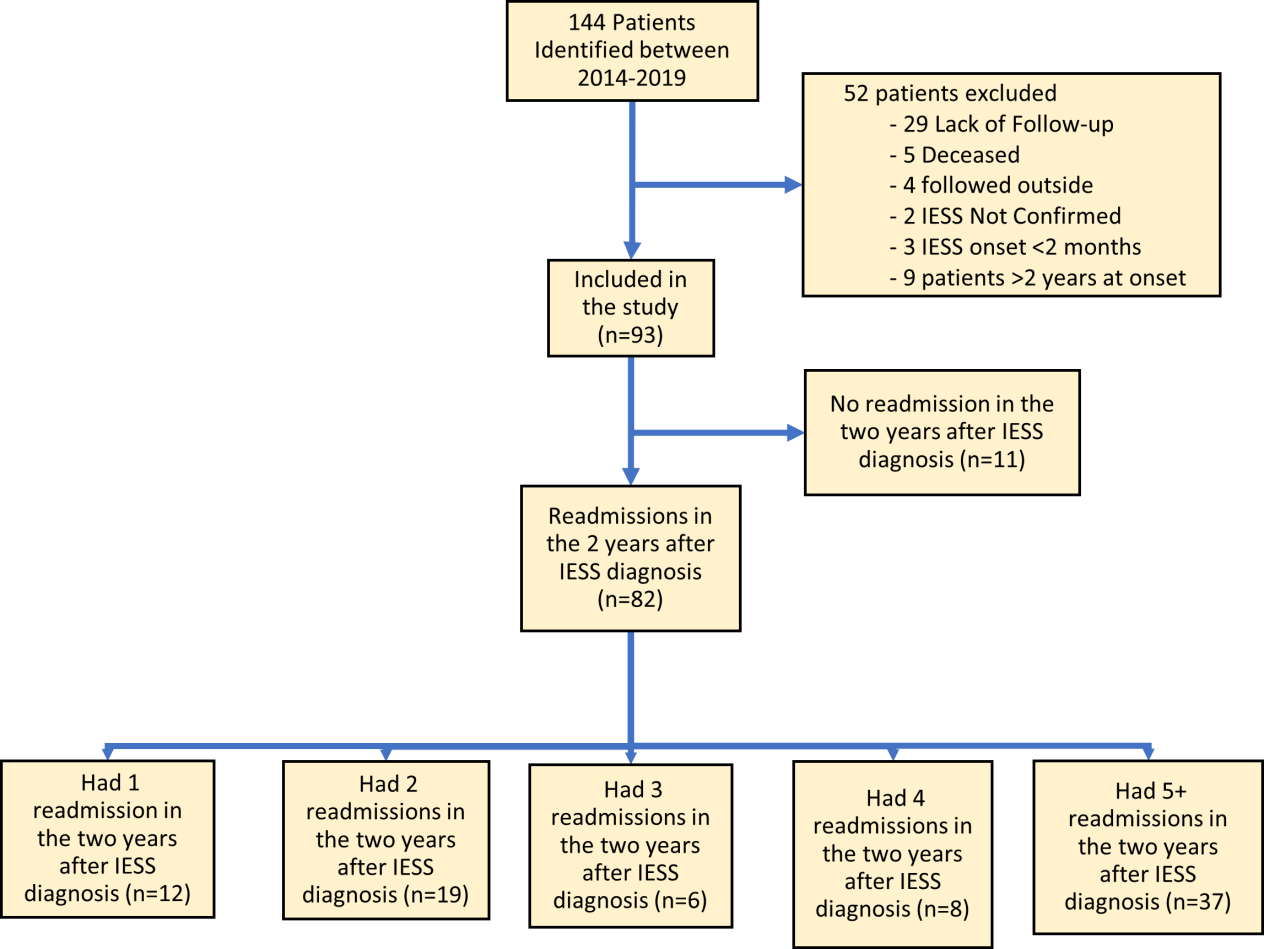
Flow diagram of the study cohort with infantile epileptic spasms syndrome (IESS).

Of 125 IESS patients identified, 93 (60% males) were eligible for the study (Figure 1). Median age at IESS onset was 6 months (interquartile range or IQR 4, 8 months) and median duration of follow‐up was 3.8 years (IQR 2.6, 5.2 years). Prior to IESS onset, seizures were present in 38 patients (41%) and developmental delay in 60 (64.5%). Etiology was known in 77 (83%), acquired in 23 (25%), and nonacquired in 54 (58%); and time to initial first‐line treatments from IESS onset was 12 days (IQR 4, 27 days). Most patients (63.4%) required multiple first‐line medications. At the last follow‐up, 43% were seizure free. Ketogenic diet was initiated in 23 (25%) at a median of 9 months (IQR 4.5, 19 months) from IESS diagnosis.

Technology use was noted in 30 patients (32%): 26 used gastrostomies; two used nasogastric tubes; one used ventriculoperitoneal (VP) shunt; and one tracheostomy. Technology use was noted prior to or around IESS onset in 15 of these 30 patients; of these, 12 had a g‐tube and one patient each had an NG‐tube (g‐tube placed later), VP shunt, and tracheostomy with g‐tube. In the remaining 14 patients, g‐tube was placed at a median 7.5 months (IQR 2, 18.7 months) from IESS diagnosis, but many of these patients had history of aspiration and/or malnutrition for several months before g‐tube placement. Multispecialty care was seen in 68% of patients. Insurance carrier was public in 35 patients (38%), private/dual in 52 patients (56%), and unknown in six patients (6%).

### 3.1 Hospital readmissions following IESS diagnosis

In the 2 years following the index admission, 11 did not have any readmissions, 24 had scheduled readmissions only (six of the 24 had exclusively protocol‐driven ones), and 58 had both scheduled and unscheduled readmissions. Overall, 82 of 93 patients (88.2%) experienced at least one further readmission and 37 of 93 (39.8%) experienced ≥5 readmissions.

**FIGURE 2:**
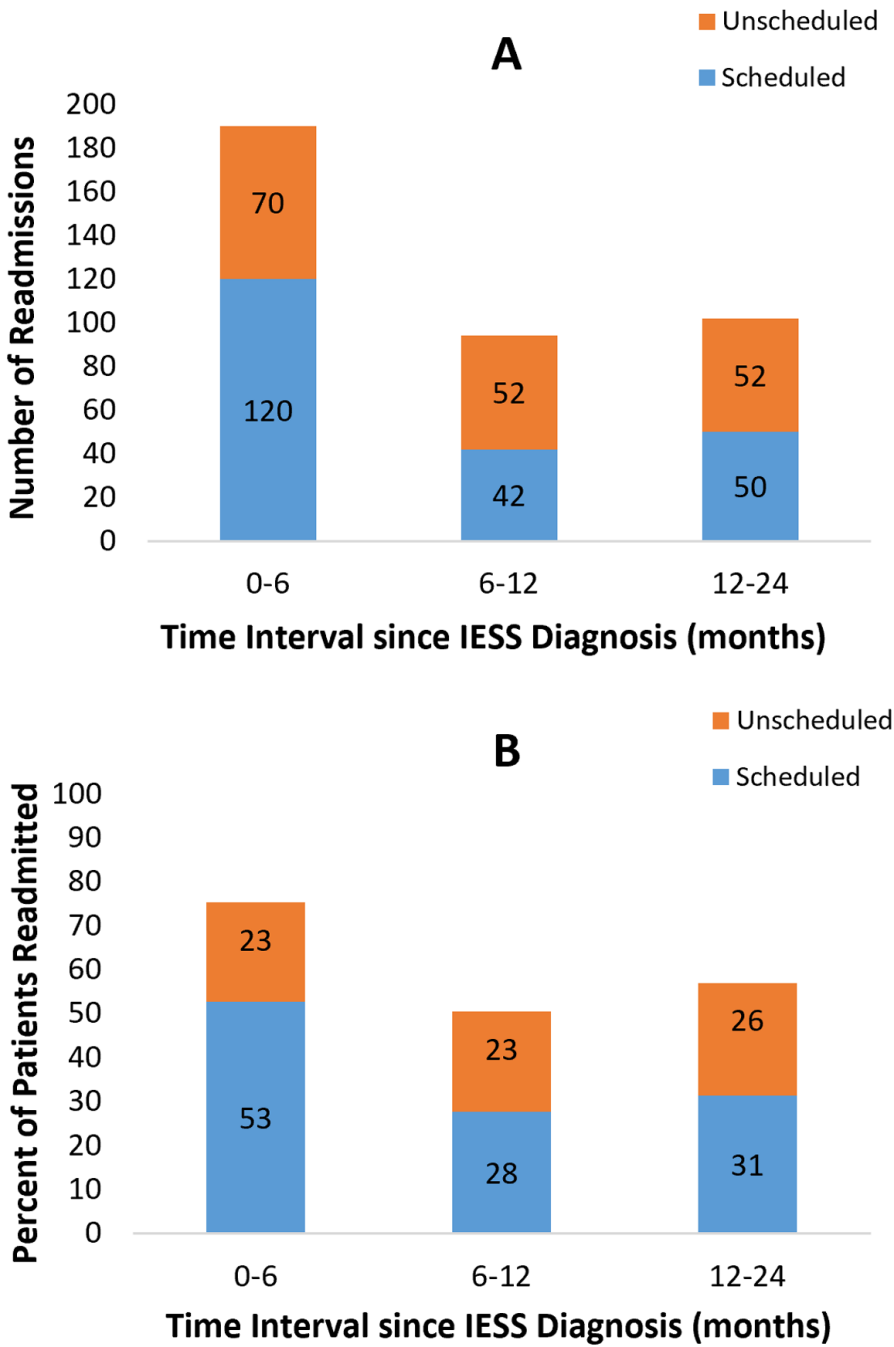
Readmissions within 2 y of infantile epileptic spasms syndrome (IESS) diagnosis. A, Number of scheduled and unscheduled readmissions within 2 y of IESS diagnosis. B, Percentage of patients readmitted within 2 y of IESS diagnosis, stratified by readmission type (scheduled and unscheduled).

**FIGURE 3:**
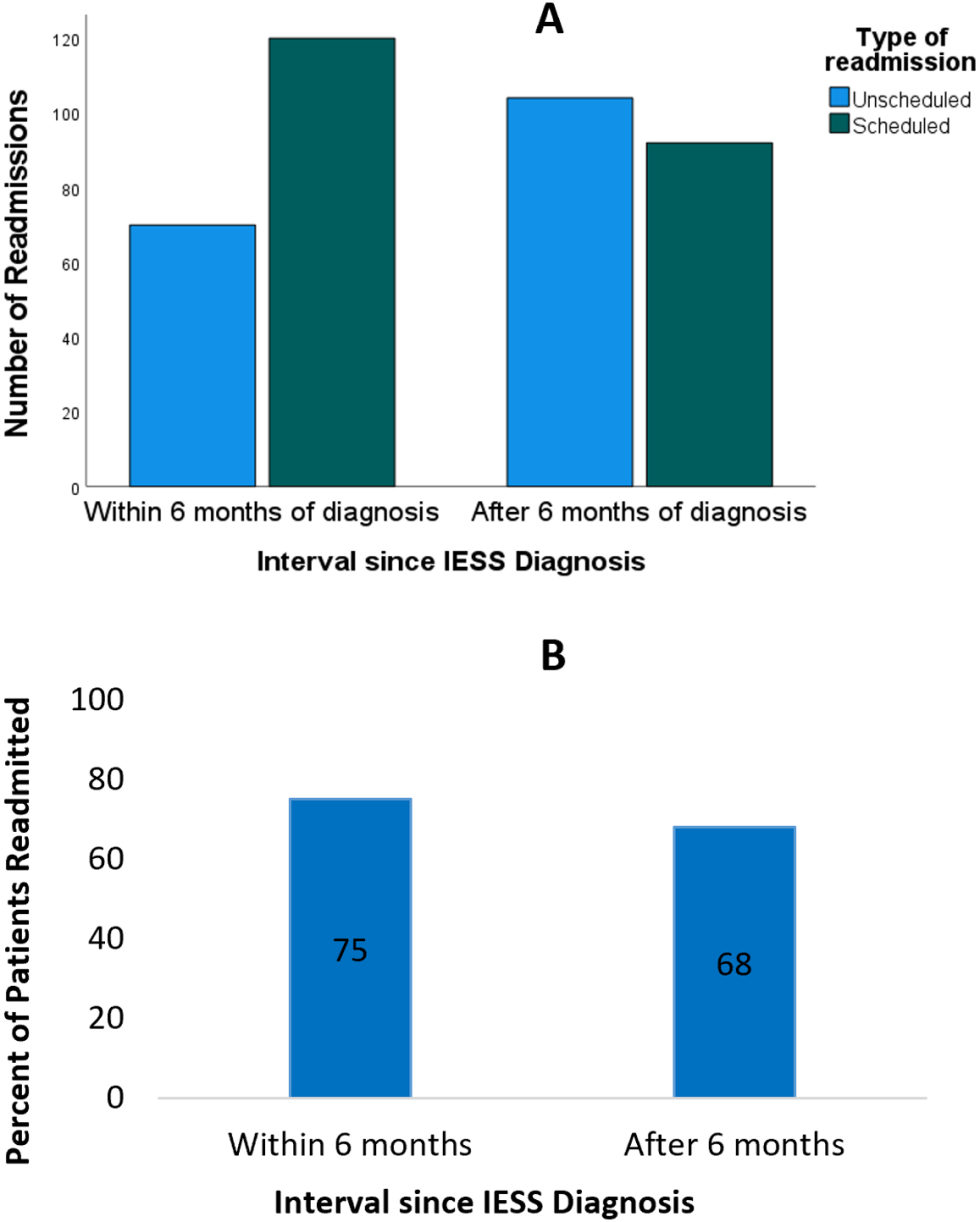
Readmissions before and after 6 mo of infantile epileptic spasms syndrome (IESS). A, Number of scheduled and unscheduled readmissions before and after 6 mo of IESS diagnosis. B, Percentage of patients readmitted before and after 6 mo of IESS diagnosis.

In the first 2 years following IESS diagnosis, there were a total of 394 readmissions, with a mean of 4.2 (SD 3.8) readmissions per patient, 220 (56%) were scheduled, and 174 (44%) were unscheduled. Readmissions occurred throughout the study period, most frequently in the first 6 months after IESS diagnosis (Figures 2A,B). Scheduled readmissions were more frequent in the 6 months following the index admission; unscheduled readmissions became more frequent after (Chi‐square test *P* ≤ 0.001; Figure 3A,B).

The subset of patients who had fewer than five readmissions had 101 readmissions, with scheduled readmissions at 66%; in patients with ≥5 readmissions (n = 293 admissions), around half (52.5%) were scheduled. Unscheduled readmissions (n = 139, 47%) were more common in patients with ≥5 admissions vs those with <5 readmissions (n = 35, Chi‐square test *P* = 0.025).

Median time between admissions was 53 days (IQR 21, 117 days) with mean length of stay of 3.5 days (SD: 5.9). Median time between readmissions was longer for those with <5 hospitalizations (57 days, IQR 21, 133 days) versus those with ≥5 readmissions (47 days, IQR 21, 101 days).

In the 2 years following IESS diagnosis, univariate analysis of all readmissions (n = 394, Table 1) showed that increased frequency of rehospitalizations was associated with use of multiple first‐line treatments (*P* ≤ 0.001), use of ketogenic diet (*P* = 0.011), use of technology assistance (*P* ≤ 0.001), and multispecialty care (*P* ≤ 0.001). Seizure freedom at last follow‐up (*P* ≤ 0.001) was associated with decreased frequency of rehospitalizations. On multivariate regression, multiple first‐line treatments (IRR‐1.59, CI‐1.15, 2.21, *P* = 0.006), use of technology assistance (IRR‐1.83, CI‐1.31, 2.55, *P* ≤ 0.001), and multispecialty care (IRR‐1.63, CI‐1.12, 2.36, *P* = 0.01) increased incidence of readmissions; seizure freedom at last follow‐up (IRR‐0.67, CI‐0.48, 0.92, *P* = 0.015), and known etiology (IRR‐0.58, CI‐0.38, 0.88, *P* = 0.011) lowered the incidence of hospital readmissions. No other variables affected the incidence of rehospitalizations (*P* > 0.05 for all).

**TABLE 1:** Regression analysis of readmissions in the 2 y following IESS diagnosis.

Variable	IRR (95% CI)		IRR (95% CI)	
	Univariate regression	*P*‐value	Multivariate regression	*P*‐value
Time to initial first‐line treatment (d)	0.998 (0.995, 1.001)	0.197	0.998 (0.996, 1.000)	0.119
Etiology, known vs unknown	1.03 (0.64, 1.67)	0.896	0.58 (0.38, 0.88)	0.011
First‐line treatment >1 vs 1	1.92 (1.33, 2.77)	≤0.001	1.59 (1.15, 2.21)	0.006
Seizure free at last follow‐up	0.55 (0.39, 0.79)	≤0.001	0.67 (0.48, 0.92)	0.015
Use of technology assistance	2.04 (1.45, 2.87)	≤0.001	1.83 (1.31, 2.55)	≤0.001
Specialists seen, ≥3 vs <3	2.06 (1.40, 3.02)	≤0.001	1.63 (1.12, 2.36)	0.01
Insurance type				
Private/dual vs public	0.80 (0.55, 1.16)	0.238	0.77 (0.57, 1.04)	0.093
Unknown vs public	0.91 (0.43, 1.93)	0.798	1.20 (0.63, 2.28)	0.583
Gender (males vs females)	0.92 (0.64, 1.33)	0.660		
Seizures prior to IESS onset	1.11 (0.77, 1.59)	0.587		
Developmental delay at IESS onset	0.87 (0.60, 1.27)	0.476		
Use of ketogenic diet	1.66 (1.12, 2.45)	0.011		
Age at IESS diagnosis (mo)	0.99 (0.94, 1.03)	0.549		

*Note*: Negative binomial regression analysis for the number of readmissions (scheduled plus unscheduled) in 2 y following IESS (infantile epileptic spasms syndrome) diagnosis (n = 79 for age of onset and for time to treatment, and n = 93 for other variables, missing numbers inputted for multiple regression). Select multivariate regression values are shown. Variables were selected by forward selection (entry significance level (SLE) = 0.2).

Abbreviations: CI, confidence interval; IRR, Incidence rate ratio.

When examining unscheduled readmissions separately (n = 174, Table 2), analysis showed results very similar to those above, with the exception that public insurance use increased unscheduled readmissions. Univariate analysis showed multiple first‐line treatments (*P* = 0.019), use of ketogenic diet (*P* = 0.010), technology assistance (*P* ≤ 0.001), multispecialty care (*P* ≤ 0.001), and public insurance (*P* = 0.035) increased unscheduled readmissions; seizure freedom at last follow‐up reduced the frequency of readmissions (*P* ≤ 0.001). On multivariate analysis, technology assistance (IRR‐2.93, CI‐ 1.74, 4.96, *P* ≤ 0.001), multispecialty care (IRR‐2.19, CI‐ 1.18, 4.05, *P* = 0.013), and use of public insurance (IRR‐1.78, CI‐1.13, 2.81, *P* = 0.013) increased the incidence of unscheduled readmissions, and seizure freedom at last follow‐up reduced the incidence of unscheduled readmissions (IRR‐0.49, CI‐0.29, 0.81, *P* = 0.006).

**TABLE 2:** Negative binomial regression analysis of unscheduled readmissions in the 2 y following IESS diagnosis.

Variable	IRR (95% CI)		IRR (95% CI)	
	Univariate regression	*P*‐value	Multivariate regression	*P*‐value
Age at IESS diagnosis (mo)	0.98 (0.91, 1.05)	0.535	0.95 (0.89, 1.01)	0.102
Etiology, known vs unknown	1.36 (0.65, 2.88)	0.415	0.51 (0.26, 1.01)	0.053
First‐line treatment >1 vs 1	1.99 (1.12, 3.55)	0.019	1.43 (0.87, 2.36)	0.159
Seizure free at last follow‐up	0.37 (0.21, 0.64)	≤0.001	0.49 (0.29, 0.81)	0.006
Use of technology assistance	3.20 (1.94, 5.27)	≤0.001	2.93 (1.74, 4.96)	≤0.001
Specialists seen, ≥3 vs <3	3.47 (1.86, 6.48)	≤0.001	2.19 (1.18, 4.05)	0.013
Insurance type				
Private/dual vs public	0.55 (0.32, 0.96)	0.035	0.56 (0.36, 0.88)	0.013
Unknown vs public	0.66 (0.21, 2.04)	0.467	1.23 (0.44, 3.42)	0.689
Gender (males vs females)	0.78 (0.44, 1.36)	0.377		
Seizures prior to IESS onset	1.18 (0.68, 2.04)	0.559		
Time to initial first‐line treatment (d)	0.99 (0.99, 1.003)	0.554		
Delays at IESS onset	1.17 (0.65, 2.08)	0.604		
Use of ketogenic diet	2.15 (1.20, 3.84)	0.010		

*Note*: Negative binomial regression analysis for the number of unscheduled readmissions in 2 y following IESS (infantile epileptic spasms syndrome) diagnosis (n = 79 for age of onset and for time to treatment, n = 93 for other variables, missing numbers inputted for multiple regression). Variables were selected by forward selection (entry significance level (SLE) = 0.2).

Abbreviations: CI, confidence interval; IRR, Incidence rate ratio.

Comparing a binary outcome (categorized as having 4 or less vs ≥5 readmissions), technology assistance increased the odds (odds ratio, OR‐ 5.40, CI‐1.59, 18.39, *P* = 0.007) for having ≥5 admissions (Table 3).

**TABLE 3:** Logistic regression analysis of subjects with five or more readmissions in the 2 y following IESS diagnosis.

Variable	OR (95% CI)		OR (95% CI)	
	Univariate regression	*P*‐value	Multivariate regression	*P*‐value
Age at diagnosis of IESS (mo)	0.91 (0.80, 1.03)	0.137	0.92 (0.79, 1.07)	0.298
Time to initial first‐line treatment (d)	0.99 (0.98, 1.00)	0.166	0.99 (0.98, 1.01)	0.305
Developmental delay at IESS onset	0.53 (0.22, 1.26)	0.147	0.54 (0.17, 1.70)	0.296
Etiology, Known vs Unknown	0.82 (0.28, 2.44)	0.722	0.44 (0.12, 1.63)	0.221
First‐line treatments, >1 vs 1	3.14 (1.22, 8.06)	0.017	2.11 (0.74, 6.00)	0.161
Use of technology assistance	3.47 (1.4, 8.60)	0.007	5.40 (1.59, 18.39)	0.007
Specialists seen, ≥3 vs <3	2.99 (1.12, 7.96)	0.029	2.87 (0.92, 8.94)	0.069
Gender (males vs females)	0.72 (0.31, 1.7)	0.457		
Seizures prior to IESS onset	1.31 (0.57, 3.04)	0.524		
Use of ketogenic diet	1.96 (0.76, 5.09)	0.165		
Seizure free at last follow‐up	0.48 (0.20, 1.14)	0.096		
Insurance type				
Private/dual vs public	0.90 (0.38, 2.15)	0.810		
Unknown vs public	1.47 (0.26, 8.27)	0.664		

*Note*: Logistic regression analysis for having five or more admissions in 2 y following IESS (infantile epileptic spasms syndrome) diagnosis (n = 37) (n = 79 for age of onset and for time to treatment, n = 93 for other variables, missing numbers inputted for multiple regression). Variables were selected by forward selection (entry significance level (SLE) = 0.2).

Abbreviations: CI, confidence interval; OR, Odds Ratio.

### 3.2 Reasons for hospitalizations

In the overall cohort, reasons for readmission included EEG monitoring (n = 199 readmissions, 50.5% including characterization of events, n = 90; protocol‐driven monitoring for confirming electroclinical remission of IS, n = 67; and other EEG monitoring including presurgical evaluation, n = 42), acute medical issues (n = 83, 21%), seizure exacerbation (n = 57, 14.5%), procedures (n = 27, 6.8%), ketogenic diet initiation (n = 15, 3.8%), and other reasons (n = 13, 3.3%).

### 3.3 Scheduled readmissions (n = 220)

The majority of scheduled readmissions (76%) were for EEG monitoring: including protocol‐driven verification of electroclinical remission of IESS (n = 67), EEG surveillance for reasons other than IESS remission (n = 30), characterization of events (n = 59), and presurgical monitoring (n = 12). Elective surgery (epilepsy surgery in four and other surgical procedures in 21) accounted for 11.3% of scheduled admissions, ketogenic diet (n = 15) for 6.8%, and other reasons (n = 12) for 5.5%.

EEG surveillance was performed for a variety of reasons other than IESS posttreatment monitoring (evaluation for EEG changes with medication or dose changes, or prior to weaning medications such as vigabatrin). EEG surveillance also occurred in cases where historically, seizures were unrecognized by family to assess seizure burden, among other reasons. Characterization of events was related to new or changing character of events or for spasms‐like spells. Other reasons included initiation of ACTH therapy, trial of BiPAP support, monitoring following postprocedural sedation, hypoxia noted during sleep study, etc.

### 3.4 Unscheduled admissions (n = 174)

Acute medical issues (n = 83) and seizure exacerbation (n = 57) accounted for 80% of unscheduled readmissions. Unscheduled EEG monitoring (n = 31) due to worsening spells or new spells with vital sign changes (sometimes with acute illness) constituted 18% of unscheduled visits. Other reasons (n = 3) included an unscheduled admission for ACTH initiation and two readmissions for unplanned procedures. Acute medical issues included respiratory illness, gastrointestinal illness, neurological symptoms (decreased mental status, irritability, and acute ataxia), dehydration, electrolyte imbalance, and hypertension, among others.

### 3.5 Change in protocolized readmissions during the study period (n = 67)

Our protocol for verifying IESS remission was changed in November 2017 to use prolonged outpatient EEGs over inpatient monitoring. Prior to the change, 56 patients were diagnosed with IESS and 37 were diagnosed after. Prior to protocol change, 62.5% (35 of the 56) patients had at least one protocolized readmission and 30% (11 of the 37) had at least one protocolized readmission after the change. This represents a significant difference in readmissions before and after the protocol modification (*P* = 0.002) and an estimated 52% decline in protocolized readmissions.

## 4 DISCUSSION

Unscheduled readmissions are considered a metric for quality of care, but both scheduled and unscheduled readmissions can add to the burden of care.^18^ While it is known that IESS patients have the highest mean annual resource utilization among epilepsy patients,^13^ there is a paucity of information on the patterns and indications for hospitalization. It is believed that most of these healthcare expenditures occur in inpatient settings, with disproportionately higher costs within the 1st year following a new epilepsy diagnosis.^18^, ^22^ We, therefore, set out to investigate the burden of all‐cause readmissions, evaluate risk factors, and contextualize reasons for readmissions in new‐onset IESS to help counsel families and understand which infants are at risk for recurrent readmissions to target potential strategies to contain hospital use.

Accordingly, we found new‐onset IESS patients have substantial inpatient resource use (40% had ≥5 readmissions within 2 years of diagnosis). This is an underestimate of the disease morbidity as we have not explored ambulatory care and emergency visits incurred by these patients. We found scheduled readmissions (56%) were more frequent than unscheduled ones (44%), especially in the first 6 months following IESS diagnosis. Our data emphasize that focusing exclusively on unscheduled readmissions would overlook many scheduled readmissions and obscure a potentially important indicator of total inpatient care delivered. Our study identified a subset at risk for frequent readmissions, namely patients with medical fragility and poor seizure control. One‐third of our patients needed technology assistance (displaying medical complexity), a key attribute specifically for intensive resource use.^18^, ^23^ Despite medical fragility, readmissions in our patients were mostly for epilepsy care (diagnostic procedures and epilepsy treatment)—which is in contrast to reports from medically fragile children^18^ (admitted for medical issues, technology‐related problems, or major surgery) or children/adults with established epilepsy,^13^, ^15^, ^24^, ^25^ where majority of resource use is for nonepilepsy care. Our study suggested at least one potential means to reduce readmissions via targeting protocol‐driven readmissions, where a significant difference in readmissions was achieved through modification of protocol.

The frequency of readmissions (all‐cause) in our patients was affected by IESS/epilepsy‐related factors (use of >1 first‐line IESS treatment, unknown etiology, or poor seizure control) and by medical complexities (technology assistance and multispecialty care). A separate analysis of unscheduled readmissions showed similar results. Additionally, public insurance use increased unscheduled readmissions in our patients, which was concordant with multicenter data involving children/infants, where public insurance use was a risk factor for readmissions.^26^, ^27^ In contrast, public insurance use was not associated with unplanned readmissions in pediatric neurology/epilepsy patients.^28^, ^29^ The discrepancy may be due to inclusion of older patients (mean age at readmission 7.6 years) or exclusion of infants in these studies. Attributes of medical complexity (poorly controlled epilepsy, need for technology use, and multispecialty care) led to increased readmissions in our study. This complements the reports of intensive inpatient resource use seen in patients with chronic care conditions (epilepsy and developmental disability included) with technology use,^18^, ^23^, ^29^ as well as in those with indwelling medical devices during the first 3 years of life.^26^, ^27^

Within 2 years of diagnosis, our patients experienced an intense cluster of readmissions (40% had ≥5 readmissions within 2 years), which may be intrinsic to the expected progression of this disorder, as ~50% of IESS patients have IESS and other seizure types occur in 30‐78% within 12‐18 months after IESS onset.^7^, ^30^, ^31^, ^32^ We consider this high volume of care experienced by our patients to be excessive. Support for our hypothesis can be drawn from readmissions data from neonatal intensive care unit (NICU) graduates (including preterm infants), infants with bronchopulmonary dysplasia, and complex‐care program patients who are known to experience frequent readmissions. In one study, 36.8% of all NICU graduates had at least one post‐NICU discharge hospitalization and another noted 25% of preterm infants were rehospitalized during the first year of life.^33^, ^34^ In comparison, 88% of our patients had at least one hospitalization following IESS diagnosis. Among complex‐care program patients, mean rehospitalization rate was 3.1 ± 2.8 over a 2‐year period.^26^ Similarly, our cohort had a mean rate of 4.2 readmissions over 2 years (2.1 per person per year). In a study of recurrent hospitalizations within Children's hospitals, Berry et al found that 2.9% patients had four or more readmissions during any 365‐day interval within the follow‐up period.^26^ In comparison, 18.2% of our patients experienced ≥8 readmissions over 2 years.

In our patients, the majority of readmissions were epilepsy related (diagnostic testing, ketogenic diet initiation, epilepsy surgery, and seizure exacerbation) similar to pediatric neurology/epilepsy readmissions, where seizures were the principal cause of unplanned or all‐cause readmissions.^28^, ^29^, ^35^

Nearly 60% of our post‐IESS diagnosis readmissions were scheduled. The majority of scheduled readmissions were for EEG monitoring, which may be inevitable when indicated for disease‐specific reasons, such as for patients with a history of subtle spasms/spasms‐like spells,^36^ monitoring for new or changing character of existing spells, or presurgical evaluation. We believe protocolized readmissions and nonprotocolized scheduled readmissions (epilepsy surgery evaluation, elective procedures, and ketogenic diet initiation) would be similar across academic institutions. Scheduled readmissions related to EEG surveillance for characterization of events may be institution/provider (preferred practices) and systems (availability of outpatient full‐day EEG monitoring) specific. Preventability of these readmissions is doubtful when scant evidence exists to guide practices (eg, ideal duration of EEG monitoring necessary to assess risk for relapse prior to ASM wean).^8^, ^37^ In such situations, neurodiagnostic testing not only depends on physician/institution‐specific practices but may also be influenced by caregiver/patient‐related factors. Despite receiving sub‐specialty ambulatory care (neurologists and epileptologists including IESS experts), 47% of the readmissions among patients with frequent readmissions were unplanned. This is likely due to the unpredictable course of epilepsy, resource availability, or low parental thresholds for presentation to the hospital.

Regardless of these challenges, we found a decline in the protocol‐driven readmissions for verification of IESS remission following protocol change. This implies that standardization of practice has the potential to prevent at least some readmissions. We have subsequently demonstrated that this modified approach along with clinical assessment was sufficient to detect treatment failure for IESS.^37^

Our study was not designed to address preventability of readmissions. Nevertheless, our study findings could rationalize other approaches to contain hospitalizations. Although not available at our hospital, prolonged outpatient EEG (4‐12 hours) has been utilized to characterize frequent spells successfully and can potentially prevent some of the scheduled readmissions.^38^ Refractory epilepsy increases readmissions. Therefore, strategic use of early resective surgery in eligible candidates (minority) and pursuing alternate treatments for nonsurgical candidates including callosotomy (treating multiple seizure types including epileptic spasms) may reduce readmissions.^39^ In medically fragile children at risk for frequent readmissions, an early referral to programs like complex‐care services with the purpose of consolidating care may reduce unnecessary hospitalizations.

### 4.1 Limitations

Smaller sample sizes from a single institution, retrospective data, and lack of a control group (infantile nonspasms epilepsy) are limitations. There may be unknown confounders. Certain attributes of our study question the generalizability and strength of our findings; single‐institution studies may be influenced by center‐specific care practices, availability of extended outpatient EEG, scope of practice (epilepsy surgery and ketogenic diet), candidate selection for technology assistance, timing of procedures, etc. Variations in readmission rates occur across adult hospitals potentially due to area differences in tendency to hospitalize or availability of hospital beds.^40^, ^41^ Prevalence/age at g‐tube placement varies, potentially reflecting differences in access to treatment and/or clinical practice.^42^ Our readmissions data reflect practice in a tertiary center from a high‐income country with adequate institutional capacity/infrastructure but likely echo practices across academic centers. Our practice of diagnostic testing related to scheduled readmissions, including protocol‐driven ones, may not be applicable in countries with different healthcare systems, developing countries, or even other institutions within the United States because of differences in healthcare practices and patient populations. However, we suspect that resource use due to unplanned readmissions would be similar, as shown by a recent study where unplanned readmissions in pediatric epilepsy did not differ by hospital type (metropolitan teaching/nonteaching/nonmetropolitan).^29^ Our findings of intense resource use in new‐onset IESS could be verified by comparing readmissions data among cohorts with IESS to other childhood epilepsies or by comparing IESS cohorts nationally and internationally.

Despite these limitations, this study adds to our body of knowledge by providing a detailed analysis of hospital resource use in new‐onset IESS. If our study findings are verified in large administrative databases, we can then investigate potential strategies to reduce hospitalizations, with the understanding that preventing readmissions is complex and may be influenced not only by demographic/patient characteristics but also by caregiver perception of discharge readiness.^43^

## 5 CONCLUSION

Our findings are helpful to counsel families regarding hospital resource use of infants with new‐onset IESS. Infants with IESS are at risk for recurrent hospitalizations within 2 years following diagnosis of IESS, mainly related to epilepsy care (diagnostic testing, treatment related, and seizure worsening). The subset of IESS patients with technology dependence and poorly controlled epilepsy are at risk for frequent readmissions. We suggest that, among the spectrum of childhood epilepsy, infants with newly diagnosed IESS should be considered “at risk” for intense healthcare utilization, especially in the first 2 years following IESS diagnosis. Since readmissions are increased by intrinsic patient characteristics, such as medical complexity or epilepsy‐related issues, the preventability of readmissions in new‐onset IESS is uncertain for most readmissions, except for protocol‐driven readmissions, which are modifiable to certain extent.

## AUTHOR CONTRIBUTIONS

**Harini**: Conceptualization; data acquisition, analysis, and interpretation; and writing—original draft and review/editing; **Yuskaitis**: data acquisition, analysis, and interpretation and critical revision of manuscript; **Singh:** data acquisition, analysis, and interpretation and critical revision of manuscript; **McHugh**: data acquisition, analysis, and interpretation and critical revision of manuscript; **Liu**: data acquisition and critical revision of manuscript; **DeLeo:** data analysis and critical revision of manuscript; **Gupta:** data acquisition and critical revision of manuscript; **Marti:** data acquisition, analysis and interpretation; and critical revision of manuscript; **Zhang:** data analysis and interpretation and critical revision of manuscript; **Libenson:** data acquisition and analysis and critical revision of manuscript; **Berry:** Conceptualization; data acquisition, analysis, and interpretation; and critical revision of manuscript;

## CONFLICT OF INTEREST STATEMENT

None of the authors has any conflict of interest to disclose. We confirm that we have read the Journal's position on issues involved in ethical publication and affirm that this report is consistent with those guidelines.

## ETHICAL APPROVAL

We confirm that we have read the Journal‘s position on issues involved in ethical publication and affirm that this report is consistent with those guidelines.

## APPENDIX A

### NEGATIVE BINOMIAL REGRESSION

We performed negative binomial regression analysis to evaluate the associations between the number of readmissions (scheduled plus unscheduled readmissions in Table 1 and unscheduled readmissions in Table 2) in the first 2 years following the diagnosis of IESS and demographic and clinical factors. We took the readmission response variable as a count variable $Y_{i}$, in which i indexed the subject number and denoted the covariate vector from the *i*th subject to be $X_{i}$. To formulate the negative binomial regression model, it was assumed that $Y_{i}$ followed a negative binomial distribution

$$P\left(Y_{i}|X_{i}\right)=\frac{\Gamma \left(y_{i}+k\right)}{\Gamma \left(k\right)\Gamma \left(y_{i}+1\right)}{\left(\frac{k}{\mu_{i}+k}\right)}^{k}{\left(1-\frac{k}{\mu_{i}+k}\right)}^{y_{i}},\;y_{i}=0,1,2,\cdots$$

where $\Gamma \left(\cdot \right)$ was the Gamma function, $\mu_{i}=E\left(Y_{i}\right)$, $\mathrm{var}\left(Y_{i}\right)=\mu_{i}+\mu_{i}^{2}/k$, and $k^{-1}$ was called a dispersion parameter.^19^ To characterize the association between $Y_{i}$ and $X_{i}$, a negative binomial regression model assumed

$$\log\left(\mu_{i}\right)=\beta_{0}+X_{i}^{\prime }\beta$$

in which $\beta_{0}$ and β denoted the regression coefficients. Negative binomial regression is an extension of Poisson regression for count data that have an extra parameter for overdispersion. Negative binomial regression models were estimated in SAS 9.4 using the procedure “genmod.”

### INCIDENT RATE RATIO

An incidence rate ratio (IRR) is the ratio of the incident rates between two different groups that we compare.^20^ Here, we compared the hospital readmission rates between two groups that were defined by a demographic or clinical factor. An IRR of <1 indicated that the incident rate was lower in one group (eg, an exposed group) compared to another group (eg, an unexposed group), and vice versa.

In the above negative binomial regression model, when a factor $X_{i}$ was binary, representing two groups (eg, $X_{i}=1$ vs $X_{i}=0$), and the model was univariate, the IRR was calculated as $\exp\left(\beta_{0}+\beta \right)/\exp\left(\beta_{0}\right)$. When a factor $X_{i}$ was continuous and the mode was univariate, the IRR was calculated as $\exp\left[\beta_{0}+\left(X+1\right)\beta \right]/\exp\left(\beta_{0}+\mathrm{X\beta}\right)$. The IRR was calculated in multivariate negative binomial regression model by controlling other factors. The procedure “genmod” in SAS 9.4 produced a confidence interval for each IRR and a *P*‐value from testing whether the IRR was equal to 1.
